# Direct electronic measurement of Peltier cooling and heating in graphene

**DOI:** 10.1038/ncomms11525

**Published:** 2016-05-10

**Authors:** I. J. Vera-Marun, J. J. van den Berg, F. K. Dejene, B. J. van Wees

**Affiliations:** 1 Physics of Nanodevices, Zernike Institute for Advanced Materials, University of Groningen, Nijenborgh 4, 9747 AG Groningen, The Netherlands; 2 School of Physics and Astronomy, The University of Manchester, Schuster Building-2.14, Manchester M13 9PL, UK

## Abstract

Thermoelectric effects allow the generation of electrical power from waste heat and the electrical control of cooling and heating. Remarkably, these effects are also highly sensitive to the asymmetry in the density of states around the Fermi energy and can therefore be exploited as probes of distortions in the electronic structure at the nanoscale. Here we consider two-dimensional graphene as an excellent nanoscale carbon material for exploring the interaction between electronic and thermal transport phenomena, by presenting a direct and quantitative measurement of the Peltier component to electronic cooling and heating in graphene. Thanks to an architecture including nanoscale thermometers, we detected Peltier component modulation of up to 15 mK for currents of 20 μA at room temperature and observed a full reversal between Peltier cooling and heating for electron and hole regimes. This fundamental thermodynamic property is a complementary tool for the study of nanoscale thermoelectric transport in two-dimensional materials.

## Abstract (web summary)

The interaction of electric and thermal transport phenomena at the nanoscale leads to Seebeck and Peltier thermoelectric effects. Here, the authors directly detect the Peltier effect in graphene, and show that it can be reversed by controlling the type and density of the majority carriers using a back gate.

## Graphical abstract

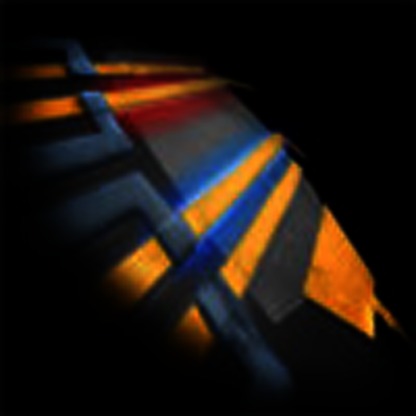

Recent advances in thermoelectrics^1^,^2^ have been fuelled by nanoscaled materials^3^,^4^, with carbon-based ones offering prospects of addressing large power density via heat management and exploiting thermoelectric effects^5^,^6^,^7^. A basic description of thermoelectrics usually involves two reciprocal processes: the Seebeck and Peltier effects. The Seebeck effect is the generation of a voltage due to a temperature difference and is quantified by the Seebeck coefficient or thermopower of a material, *S*=−Δ*V*/Δ*T*, used for temperature sensing in thermocouples. Graphene^8^,^9^,^10^ has been shown theoretically^11^,^12^,^13^ and experimentally^14^,^15^,^16^ to have a large and tunable *S* up to ±100 μV K^−1^ at room temperature, due to its unique electronic band structure and electrostatic tunability of the density and polarity of its charge carriers. In contrast, the Peltier effect describes the heating or cooling of a junction between two different materials when an electric charge current is present. It is quantified by the Peltier coefficient *Π*, which can be understood as the heat transported by thermally excited charge carriers. The Peltier effect is a reversible thermodynamic phenomenon that depends linearly on the current, so it is fundamentally different from the irreversible Joule heating^17^. More importantly, as both thermoelectric coefficients are related by the second Thomson relation^18^ *Π*=*ST*, where *T* is the reference temperature, it follows that in graphene also the Peltier coefficient *Π* (and its associated cooling or heating action) can be controlled in both magnitude and sign. Until now, one study managed to detect Peltier heat in a graphene–metal junction^19^, nevertheless without demonstrating any significant modulation nor reversal of the Peltier effect with carrier density, and involved a complex scanning probe microscopy technique.

This work presents a direct and quantitative electronic measurement of Peltier cooling and heating, in both single layer (SL) and bilayer (BL) graphene, demonstrating full modulation of the Peltier effect via electrostatic gating. We use nanoscale thermocouples for a sensitive and spatially resolved thermometry of the Peltier electronic heat evolved or absorbed at a graphene–metal junction. The results are consistent with the reversibility and electron-hole symmetry expected for the linear response of the Peltier effect. Furthermore, we probe both the local temperature change on the junction where the Peltier effect is induced, as well as in another junction some distance away. We successfully describe the observed temperature profile in the device using a simple one-dimensional model.

## Results

### Device architecture

**Figure 1:**
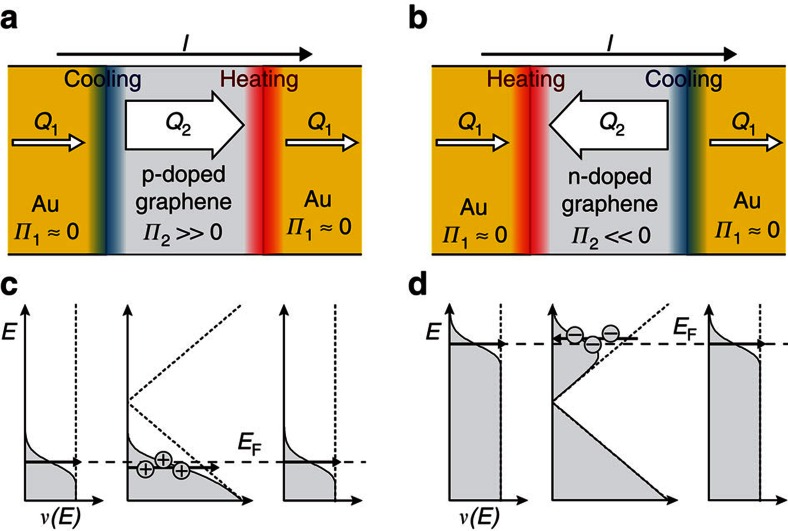
Depiction of the Peltier effect at a graphene–Au interface. (**a**,**b**) Graphene (grey) has a larger Peltier coefficient *Π* than Au (yellow) and thus a current *I* can carry more heat in graphene (*Q*_2_) than in Au (*Q*_1_). As charge flow is conserved, heat is accumulated (red) or absorbed (blue) at the interfaces. (**c**,**d**) The large *Π* in graphene is caused by the strong variation of the density of states *ν*(*E*) around the Fermi energy *E*_F_, lowering or elevating the average energy of the thermalized carriers (indicated by the black arrows). The effect reverses when tuning the carriers from (**a**,**c**) the hole regime to (**b**,**d**) the electron regime.

We induced the Peltier effect by sending a charge current through a graphene–Au metal junction (Fig. 1). With the current *I* directed from graphene to Au, the evolution of the Peltier heat at the junction is then given by 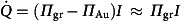, as for most carrier densities |*Π*_gr_|⩾*Π*_Au_ (refs 14, 15, 16, 20). In the hole regime *Π*_gr_>0, which corresponds to Peltier heating of the junction, as depicted in Fig. 1a. Reversely, a junction where *I* goes from Au into graphene has a cooling rate of the same magnitude. Finally, in the electron regime *Π*_gr_<0, reversing the effects of cooling and heating (Fig. 1b).

**Figure 2:**
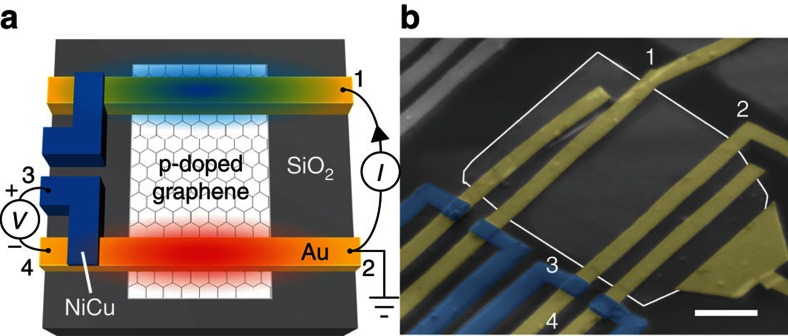
Device architecture and measurement configuration. (**a**) Schematic of the measurement geometry. A graphene flake (white) on a Si/SiO_2_ substrate is contacted with Au leads (yellow). NiCu leads (blue) form thermocouples to probe the temperature of the graphene–metal interface. We define the current as sent from contact 1 to 2 and probe the thermocouple at contacts 3 and 4. (**b**) Coloured electron micrograph of an actual device. Scale bar, 1 μm. The graphene flake is outlined in white for clarity.

To probe the electronic temperature of the Peltier junction we used nanoscale NiCu/Au thermocouple junctions (*S*_NiCu_≈−30 μV K^−1^ and *S*_Au_≈2 μV K^−1^, see Methods, Supplementary Fig. 1 and Supplementary Note 1), placed outside the graphene channel but in close proximity to the Peltier junction. The thermocouple builds up an open circuit potential *V*_tc_=(*S*_NiCu_−*S*_Au_)Δ*T*=*S*_tc_Δ*T* (between contacts 3 and 4 in Fig. 2) when a temperature difference Δ*T* exists at the thermocouple junction with respect to the reference temperature *T*. This sensitive nanoscale thermometry can detect temperature changes in the mK regime^21^. Most importantly, this approach does not require any charge current present in the thermocouple detection circuit, making it compatible with the requirement of applying a current through the graphene–metal junction for the generation of the Peltier effect. This is in contrast to the resistive thermometry used in the standard architecture for the Seebeck effect^14^,^15^,^16^ where a sensing current along the resistor is needed.

### Themoelectric measurements

For the electrical generation of the Peltier effect we applied a low frequency AC current *I* of up to 20 μA to the graphene–metal junction (between contacts 1 and 2 of Fig. 2) and used a lock-in technique to measure the thermocouple voltage. With this technique, we can distinguish between Peltier (∝*I*) and higher-order contributions such as Joule heating (∝*I*^2^) by separating the first harmonic response to the heat modulation at the junction. From the second harmonic, we estimate that Joule heating at 20 μA is ∼10 mK at 300 K, similar to the Peltier cooling and heating. Our measurement scheme allows us to single out the Peltier component, excluding all other possible sources from the measured signal (see Methods, Supplementary Fig. 2 and Supplementary Note 2) and realizing a complementary tool for the study of nanoscale thermoelectric transport in two-dimensional materials. Here we quantify the Peltier signal by normalizing the voltage generated at the nanoscale thermocouple by the current driving the Peltier junction, *V*_tc_/*I*. Thus, our measurement scheme consists of a graphene channel circuit that generates a heat current via the Peltier effect and a nonlocal detector circuit that converts this heat current back into a charge voltage via the Seebeck effect^20^.

**Figure 3:**
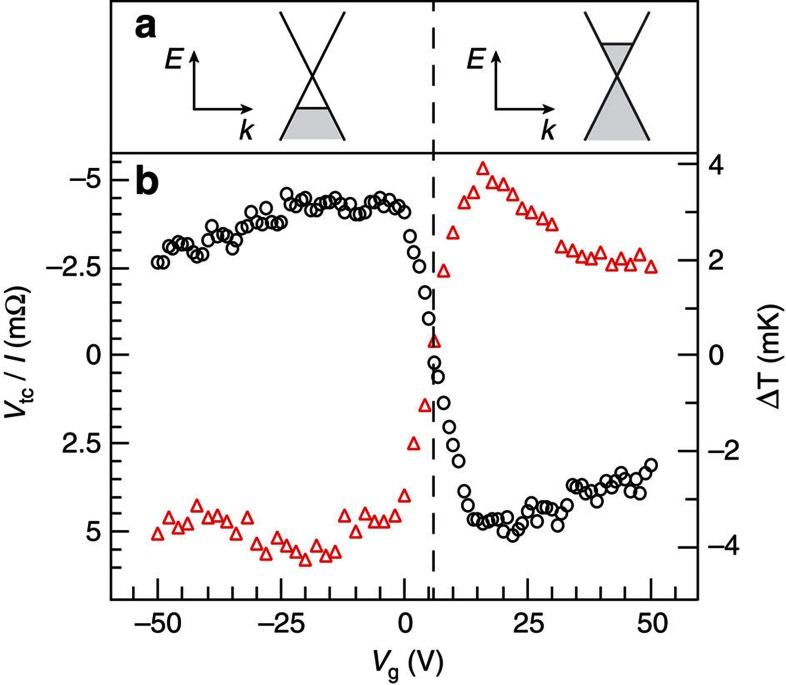
Peltier effect in a forward and reverse biased graphene–Au interface. (**a**) Schematic of the linear dispersion for p-doped (left) and n-doped (right) SL graphene. (**b**) Measurement of the thermocouple signal *V*_tc_/*I* as a function of backgate voltage, for *I*=20 μA, with the configuration shown in Fig. 1a (black circles). *V*_tc_ is converted into a temperature by using the Seebeck coefficient of the thermocouple *S*_tc_, as shown in the right axis. The hole regime shows heating, whereas the electron regime shows cooling. The vertical dashed line indicates the position of the Dirac point. For a reversed configuration of the current source (red triangles) the effects of cooling and heating are reversed.

**Figure 4:**
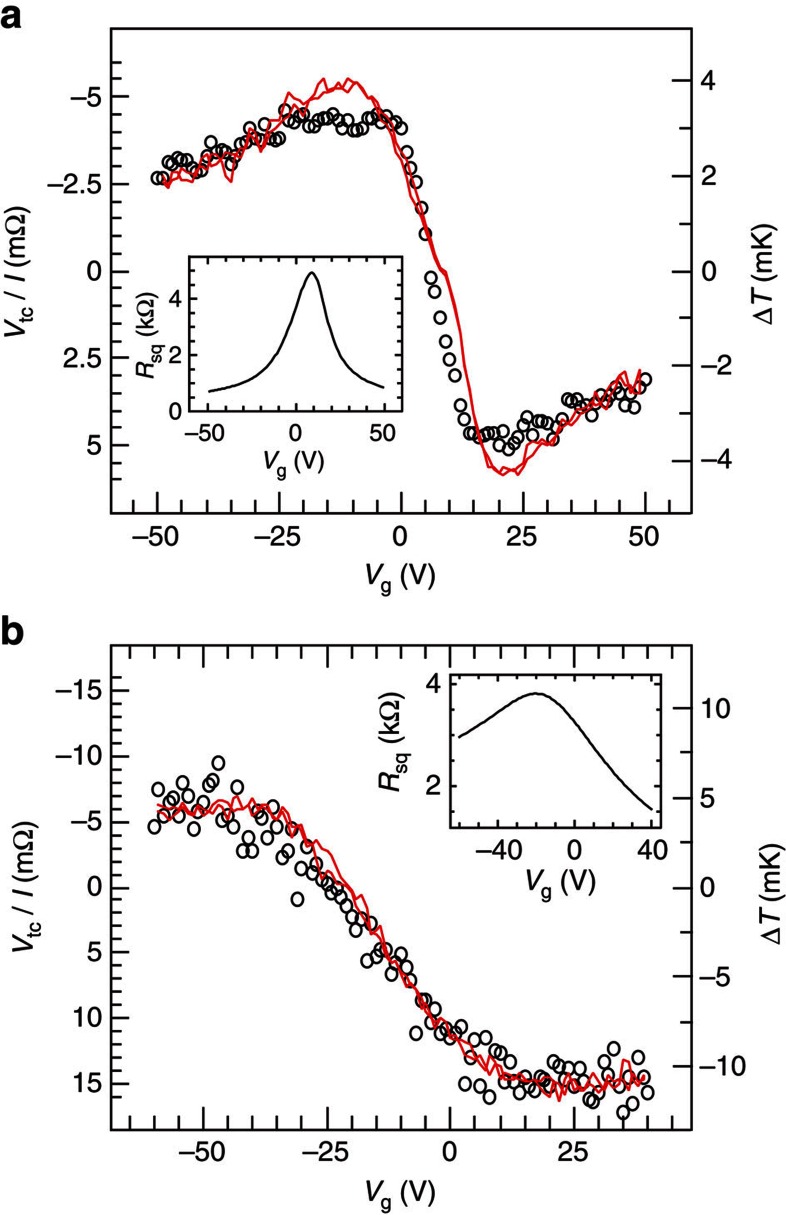
Comparison of the Peltier effect in SL and BL graphene. (**a**) Comparison of Peltier measurement for SL graphene (black circles), with a calculation of the Peltier effect line shape (red lines) derived from charge transport (inset, black lines), using equations (1) and (2). (**b**) Similar comparison for BL graphene, with a calculation using equations (1) and (3).

We observed a modulation in the thermocouple signal *V*_tc_/*I*≈10 mΩ when changing the carrier density in SL graphene with the use of a backgate potential *V*_g_ (see Fig. 3). This corresponds to a modulation of the Peltier coefficient *Π*_gr_. First, we consider the measurement configuration shown in Fig. 2a, with the current direction defined from graphene to metal and the thermocouple electrode grounded. For this configuration we observed a clear change in polarity in *V*_tc_/*I*, indicating a reversal of the Peltier effect between heating (*V*_g_<5 V) and cooling (*V*_g_>5 V). This is consistent with the location of the charge neutrality (Dirac) point (see inset in Fig. 4a) and with the symmetric band structure in graphene. In addition, we consider a reversed configuration with connections to the current source exchanged, such that now the current direction is defined from metal to graphene and the electrode grounded is not the one with the measured thermocouple. This leads to a mirroring of the signal around *V*_tc_=0, consistent with the reversible nature of the Peltier effect. The resulting temperature modulation Δ*T*=*S*_tc_*V*_tc_ due to the Peltier effect at the graphene–metal junction was ≈8 mK (Fig. 3, right axis).

## Discussion

For a better understanding of the data we calculate *Π*_gr_ from independent charge transport measurements and then use a simple heat balance to describe the temperature modulation Δ*T* at the graphene–metal junction. We relate Δ*T* to the Peltier heating and cooling rate 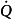 via,


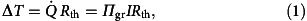


with *R*_th_ the thermal resistance sensed by the Peltier heat source at the junction, given by the heat transport through the graphene channel and Au electrode, plus the heat flow away to the Si substrate via the SiO_2_ insulator. As in graphene the thermal conductivity *κ*_gr_ is dominated by phonons^6^,^7^, *R*_th_ is a constant scaling parameter independent of *V*_g_. In contrast, *Π*_gr_ dominates the line shape of the response. To calculate *Π*_gr_, we employ the semi-classical Mott relation^22^ together with the density of states for SL graphene, *ν*(*E*)=2*E*/*π*(*ℏv*_F_)^2^, to obtain the thermopower^14^. Considering the second Thomson relation, this leads to:


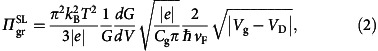


with *k*_B_ the Boltzmann constant, *e* the electron charge, *ℏ* the reduced Planck's constant, *v*_F_ the Fermi velocity, 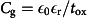 the gate capacitance per unit area, with *t*_ox_ the SiO_2_ thickness, 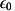 and 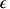 the free-space and relative permittivities, respectively, and *G* the measured charge conductivity from the Dirac curve.

Figure 4a compares the line shape of Δ*T* estimated using equations (1) and (2) with the Peltier measurement from Fig. 3, where we fit the thermal resistance parameter *R*_th_ with a value to allow a direct comparison at large *V*_g_. The good agreement between the two only deviates near the peak in the hole regime. This is because the Peltier effect probes the local density of states at the graphene–metal junction. Therefore, it is much more sensitive to doping from the contact than the Dirac curve (shown in the inset) of the graphene region in between the contacts, which only shows a small electron-hole asymmetry^23^. This observation is consistent with our previous work on nonlinear detection of spin currents in graphene^24^, where we have observed a similar modulation in the line shape of a thermoelectric-like response due to contact doping.

Figure 4b shows measurements of Peltier cooling and heating in a BL graphene device. We observed the characteristic transition from heating in the hole regime towards cooling in the electron regime, with a temperature modulation of ∼15 mK. The transition, located at *V*_g_≈−25 V, correlates with the observed charge neutrality point at *V*_D_=−20 V in the charge transport (see inset). The nonmonotonic behaviour of the Peltier signal is visible for the electron regime, but the parabolic dispersion in BL graphene leads to a broader Peltier curve than for SL graphene. We apply a similar approach as before, to estimate the temperature at the BL graphene–metal junction. Here we use the density of states of BL graphene, *ν*(*E*)=2*m*/(*πℏ*^2^), together with the semiclassical Mott relation, leading to:


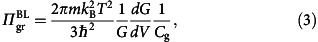


with *m*≈0.05*m*_e_, where *m*_e_ is the free electron mass^10^. The modelled line shape, shown in Fig. 4b, is again scaled by fitting the thermal resistance parameter *R*_th_. We observed an overall agreement between the data and the model, with a lower Peltier signal in the hole regime being consistent with the broader Dirac curve.

**Figure 5:**
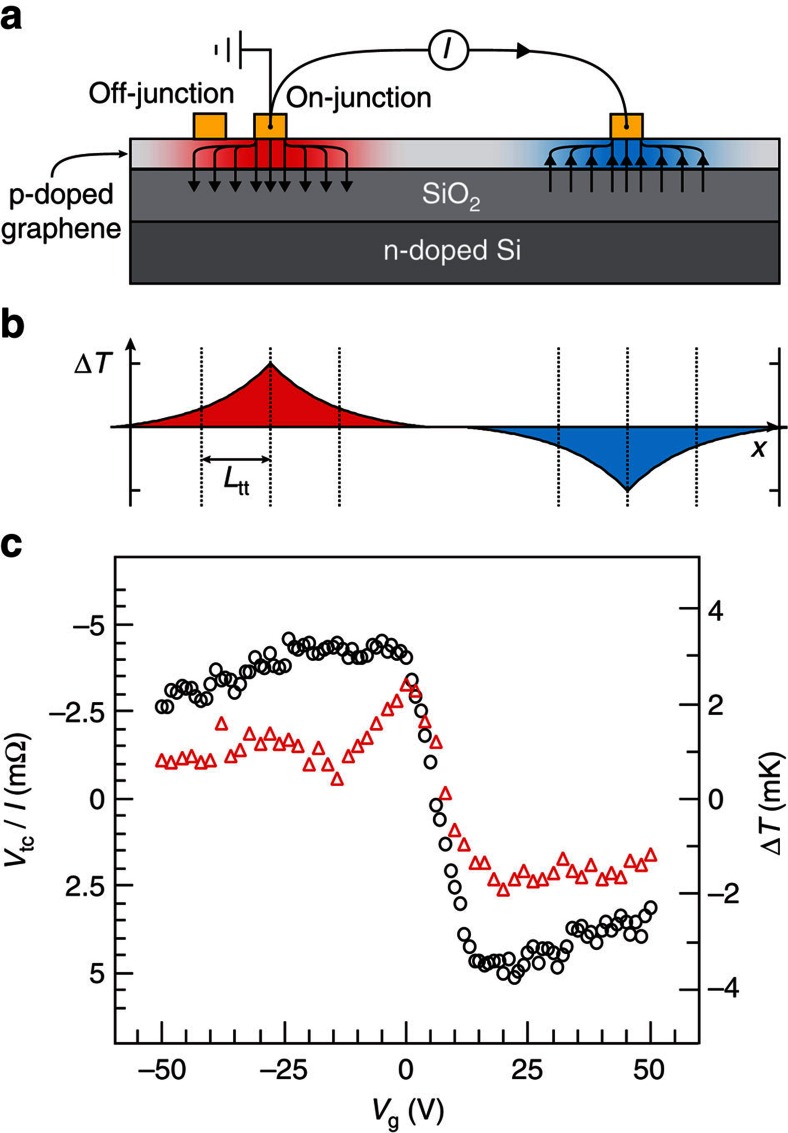
Temperature profile and off-junction measurement. (**a**) Schematic cross-section of thermal transport due to Peltier effect (not to scale). (**b**) Temperature profile in graphene, with characteristic length *L*_tt_ mentioned in the text. (**c**) Peltier measurement in SL graphene for a thermocouple contacting the graphene–metal junction where the Peltier effect is induced (black circles), and for a thermocouple on an adjacent contact 280 nm away from the junction (red triangles).

A quantitative understanding requires estimating the magnitude of the thermal resistance *R*_th_. Given the geometry of the devices, this usually involves detailed numerical thermal models. To offer physical insight we use a simple one-dimensional model for the heat flow along graphene, with a non-conserved heat current as it flows away via the SiO_2_ insulator into the Si substrate acting as a thermal reservoir (Fig. 5a). Here we introduce the concept of a thermal transfer length *L*_tt_, defined as the average distance heat flows along the graphene channel (Fig. 5b). It is given by^25^ 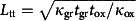, with *κ*_gr_ (*κ*_ox_) the thermal conductivity and *t*_gr_ (*t*_ox_) the thickness of graphene (SiO_2_). Considering the thermal conductivity *κ*_gr_=600 W m^−1^ K^−1^ for SL graphene supported on a Si/SiO_2_ substrate^26^, which is reduced from its intrinsic value due to substrate coupling, we estimate *L*_tt_≈320 nm. The small value indicates that the temperature modulation due to the Peltier effect diffuses laterally a short distance from the contact. With this characteristic length, we can readily estimate the thermal resistance of a heat transport channel in analogy to the study of spin resistance^24^ (see Methods). The estimated *R*_th_≈1 × 10^5^ K W^−1^ from the one-dimensional description serves as an upper limit for the thermal resistance, in agreement with the one order of magnitude lower scaling parameter *R*_th_≈1 × 10^4^ K W^−1^ used for fitting the modelled curves in Fig. 4.

Finally, we mention two other tests that shed further light on the Peltier origin of the signal. First, Fig. 5c compares the temperature measurement at the Peltier junction in SL graphene with a new measurement where we probed another thermocouple in an adjacent contact, separated by a distance *L*=280 nm. Thus, we can validate the estimated *L*_tt_, as the new measurement should be lower by a factor 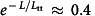. The result in Fig. 5b agrees with this estimation. A second test consisted of repeating the measurement from Fig. 3 at 77 K, where we expect the temperature dependence to be dominated by the scaling of the Peltier coefficient, *Π*_gr_∝*T*^2^. The result, a signal one order of magnitude lower (Supplementary Fig. 3 and Supplementary Note 3) further confirms the thermoelectric origin of the response.

Direct measurement of the Peltier effect offers a complementary approach to the study of nanoscale thermoelectric transport in graphene and related two-dimensional materials. Besides providing additional control in electronic heat management at the nanoscale^5^,^6^,^7^, quantifying the Peltier effect is useful for studying fundamental thermodynamic relations. In particular, nonlocal measurements involving heat, spin and valley degrees of freedom^24^,^27^,^28^,^29^,^30^ have ignored the possibility of a linear Peltier contribution, which will always be present, even without an external magnetic field.

## Methods

### Sample fabrication

SL and BL graphene flakes were mechanically exfoliated on a Si/SiO_2_ substrate. To fabricate the device geometry shown in Fig. 2 we used electron beam lithography. First, we deposited using electron beam evaporation Ti (5 nm)/Au (45 nm) electrodes to create ohmic contacts to graphene. The Si substrate was used as a backgate electrode to control the carrier density through a SiO_2_ dielectric of thickness *t*_ox_=500 nm. Next, after a short cleaning step of the Au surface using Ar ion beam etching, we deposited using sputtering NiCu electrodes to form nanoscale NiCu/Au thermocouples in close proximity to the graphene–metal Peltier junction. We selected NiCu for its large Seebeck coefficient of *S*_NiCu_≈−30 μV K^−1^ (see Supplementary Fig. 1 for an independent measurement) to be used as a thermometer^21^ and Au as a contact electrode for the Peltier junction because of its small thermoelectric response (|*Π*_gr_|⩾*Π*_Au_) with a Seebeck coefficient of only^20^ *S*_Au_≈2 μV K^−1^. All measured devices (two BLs and one SL) showed consistent results and had typical dimensions of a few micrometres. In this study, we present results for a SL with a channel width of 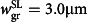 and a BL with 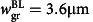.

### Peltier measurement

The measurement of the Peltier effect in a graphene transistor involved the challenge of a sensitive and local thermometry, for which the nanoscale thermocouples were developed. To achieve sub-mK resolution we required the measurement of thermocouple responses in the order of *V*_tc_/*I*≈1 mΩ. Therefore, we established a careful measurement protocol to differentiate the Peltier response from extrinsic effects.

We applied low amplitude AC currents *I*≤20 μA to the graphene–metal junctions (between Au contacts 1 and 2 of Fig. 2) to keep the response in the linear regime. We used a lock-in technique to separate the first harmonic response to the heat modulation at the graphene–metal junction, to determine the contribution due to Peltier cooling and heating (∝*I*).

Owing to the finite common mode rejection ratio of the electronics, a local resistance of order 1 kΩ can lead to a response of the order of 10 mΩ, even for a differential nonlocal measurement. Therefore, all measurements were performed for the sensing configuration shown in Fig. 2, where we measured *V*_34_=*V*_3_−*V*_4_, and then repeated for a configuration where the voltage detectors were reversed, *V*_43_. This allowed us to extract the true differential mode signal *V*_DM_=(*V*_34_−*V*_43_)/2 and to exclude common mode contributions (see Supplementary Fig. 2).

The frequency *f* of the AC current was kept low to avoid contributions due to capacitive coupling, for example, between the leads and the backgate. To exclude this contribution, the Peltier lineshape was checked at several frequencies, with a consistent lineshape typically observed for *f*≤10 Hz. All measurements shown here are for *f*≤3 Hz. Small offsets of about 1 mΩ were corrected by measuring the frequency dependence in the range 0.5–5 Hz and extrapolating to 0 Hz (see Supplementary Fig. 2).

Finally, we directly measured the Seebeck coefficient of the NiCu/Au thermocouples via an independent device geometry (see Supplementary Fig. 1). The result, *S*_tc_=*S*_NiCu_−*S*_Au_=−27 μV K^−1^, is consistent with previous estimations^21^ and was used to convert the thermocouple signal to a temperature modulation via Δ*T*=*S*_tc_*V*_tc_.

### Thermal model

Here we describe a simplified heat transport model that allows us to estimate the thermoelectric Peltier response and understand its dependence on material parameters analytically. In this model we consider graphene as a one-dimensional diffusive heat transport channel, where the Peltier junctions are treated as point sources for heat currents and the SiO_2_ substrate acts as a path for heat flow from the graphene into the underlying Si thermal bath. The one-dimensional diffusive description is appropriate, as the SiO_2_ insulator, with *t*_ox_=500 nm, dominates the thermal transport and is smaller than the width *w*_gr_ of the graphene channel^25^. Treating the junctions as point sources and disregarding current crowding effects is valid because of the narrowness of the Au contacts, where *w*_Au_≤500 nm≈*L*_ct_, with *L*_ct_ being the transfer length for charge transport between the contacts and graphene^19^.

Notably, the model is analogous to models commonly used to describe diffusive spin transport^24^. Therefore, it offers physical insight regarding the magnitude of the thermal resistance seen by the Peltier junction, which, in analogy to the study of spin resistance, yields


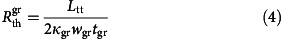


for heat transport along the graphene channel. Here, 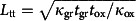 is the thermal transfer length introduced in the main text, with *κ*_ox_=1 W m^−1^ K^−1^ and *κ*_gr_=600 W m^−1^ K^−1^ (ref. 26). This heat balance only takes into account heat transport along graphene and the substrate. In practice, there is also transversal heat dissipation through the leads. To account for the latter, we apply the same model above to heat transport across the Au leads and calculate an analogous thermal resistance 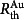. For typical device geometries, we obtain 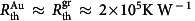. We then estimate the total thermal resistance 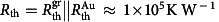, to account for the heat balance of equation (1).

It is noteworthy that the distance between the NiCu/Au thermocouples and the graphene channel (≈500 nm) is smaller than the thermal transfer length of the Au leads, 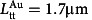, as Au is a good thermal conductor (*κ*_Au_=127 W m^−1^ K^−1^). Therefore, there is only a correction of 30% to account for the detection efficiency of the thermocouples. We note that this model can only obtain an order of magnitude estimate. It serves as an upper limit for the actual thermal resistance, because it neglects increased lateral heat spreading near the graphene edges and the finite width of the contacts. Finally, using the Wiedemann–Franz law we calculate that electrons only contribute up to 5% to the thermal conductivity of supported graphene, validating our treatment of 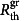 as a constant parameter.

## Additional information

**How to cite this article:** Vera-Marun, I. J. *et al*. Direct electronic measurement of Peltier cooling and heating in graphene. *Nat. Commun.* 7:11525 doi: 10.1038/ncomms11525 (2016).

## Supplementary Material

Supplementary Information Supplementary Figures 1-3, Supplementary Notes 1-3 and Supplementary References
