# Supplementary material for: Direct electronic measurement of Peltier cooling and heating in graphene
Source: Nat Commun. 2016 May 10;7:11525. doi: 10.1038/ncomms11525 (PMC4866327; doi:10.1038/ncomms11525)
Supplement: Supplementary Information — Supplementary Figures 1-3, Supplementary Notes 1-3 and Supplementary References [file ncomms11525-s1.pdf]

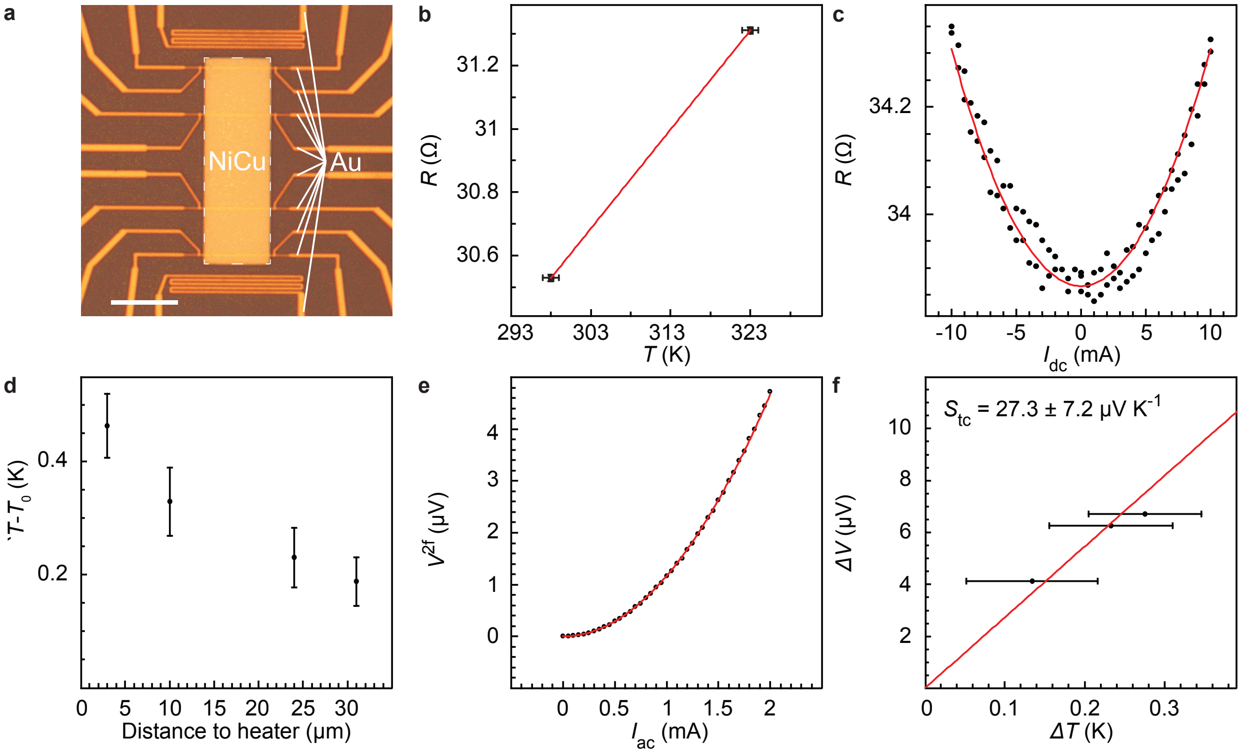

Supplementary Figure 1. **Thermocouple calibration.** (a) Micrograph of the geometry for measuring the Seebeck coefficient $S_{\mathrm{tc}} = S_{\mathrm{NiCu}} - S_{\mathrm{Au}}$. The device consists of a NiCu channel with Au bar electrodes. The microfabricated Au heater creates a temperature gradient which we mapped using the 4-probe resistance of the Au bars. The Au bars were also used as contacts to measure the Seebeck voltage of the NiCu/Au thermocouple. Scale bar is 10 µm. (b) Resistance of a Au bar versus global temperature (black squares). Error bars are the s.d. in the global temperature measurement. For this bar, the linear fit (red line) gives a thermistor sensitivity of 31 ± 3 mΩ K^{-1}. (c) Dependence of the resistance of a Au bar to a dc current I_{dc} applied through the microfabricated Au heater (black circles). The quadratic fit (red line) gives the thermistor response in units of Ω A^{-2}. (d) Temperature profile along the NiCu film extracted at each Au bar, for a fixed $I_{\mathrm{dc}} = 2$ mA. Error bars are the s.d. in the temperature at each bar propagated from the analysis of fits as those in (b) and (c). (e) Seebeck voltage along the NiCu channel due to an ac heater current (black circles), showing a quadratic ($\propto I^2$) dependence and no voltage offset at $I_{\mathbf{ac}} = 0$ mA (red line). (f) Seebeck voltage ΔV against the temperature ΔT (black circles) between pairs of Au bars shown in (d). The linear fit (red line) gives a Seebeck coefficient of $S_{\mathrm{tc}} = -27.3 \pm 7.2$ µV K^{-1}.

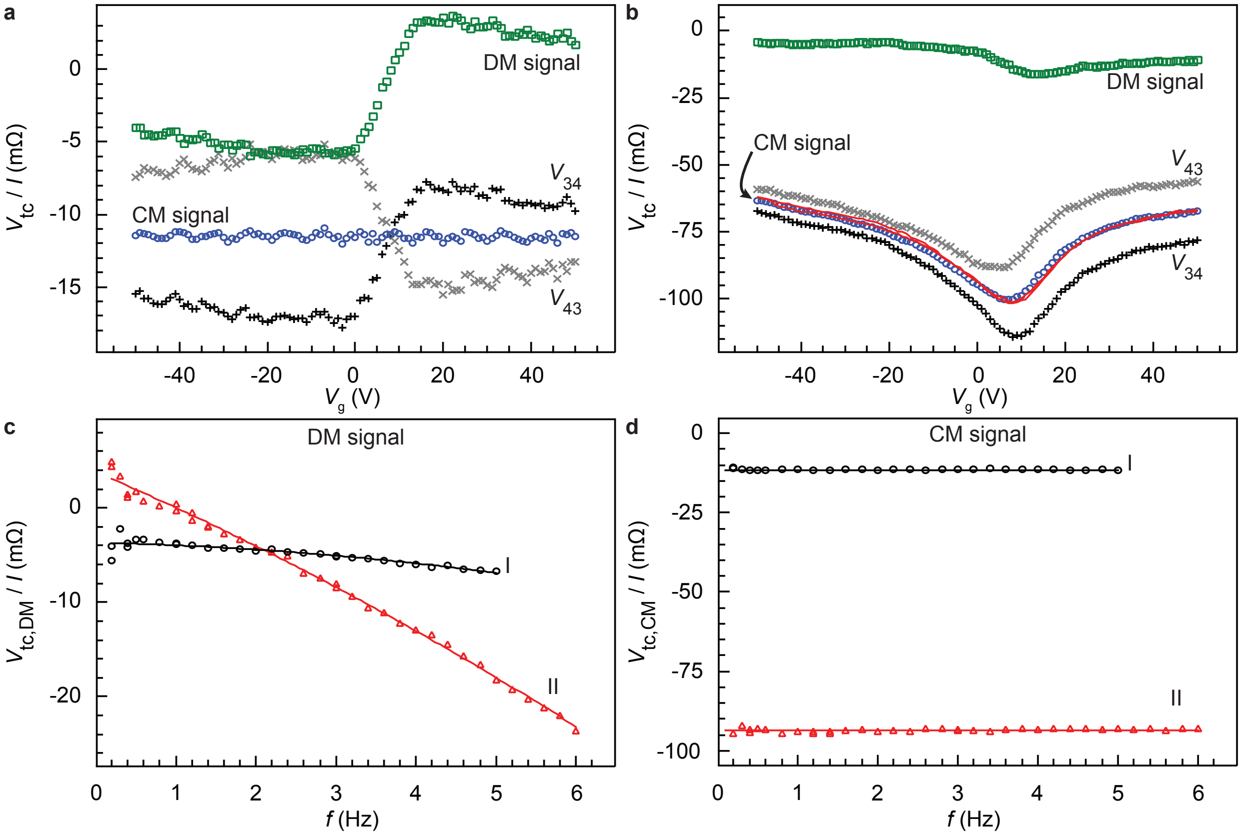

Supplementary Figure 2. **Analysis of the thermocouple signal.** (a) Gate dependence of the thermocouple response when a current $I = 20$ µA is applied to the Peltier junction, in the standard current configuration (I) as shown in Fig. 2. The figure shows the raw data, with V_{34} defined as having the voltage probes plus and minus as in Fig. 2 of the main text (black pluses) and V_{43} for the voltage probes reversed (gray crosses). The blue circles show the common mode (CM) and the green squares show the differential mode (DM). This measurement was performed at $f = 3$ Hz. (b) Similar measurements for the reversed current source configuration (II) with different grounding, as described in the main text. In this case, the CM signal is gate dependent due to the contribution of the graphene channel. For a consistency check, we also obtained the source of the CM signal by measuring the resistance R_3 as described in the Supplementary Note 1 and multiplying this with the CMRR of the electronics (red line). This measurement was performed at $f = 1$ Hz. (c) Frequency response of the DM mode at $V_g = 0$ in configuration I (black circles) and II (red triangles). The solid lines show the fitting (second order polynomial) functions used to extrapolate to 0 Hz. (d) DM mode for both configurations at $V_g = 0$, which is frequency independent and given by CMRR $\times$ R_3 with R_3 equal to 1.3 kΩ (configuration I, black circles) and 10.5 kΩ(configuration II, red triangles).

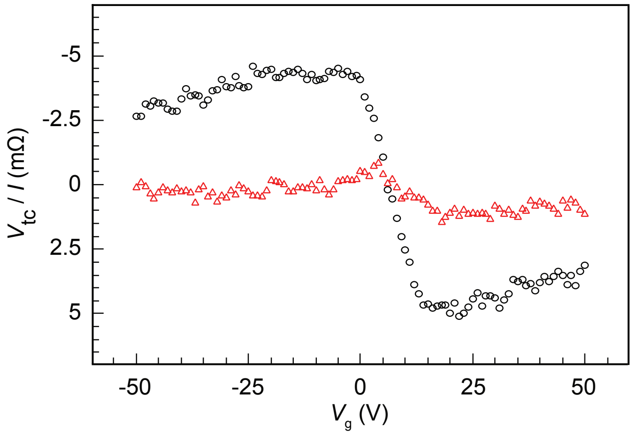

Supplementary Figure 3. **Peltier effect at low temperature.** (a) Comparison of Peltier heating and cooling at room temperature (black cirles, as in Fig. 3 of the main text) and at 77 K (red triangles).

SUPPLEMENTARY NOTES

Supplementary Note 1. Calibration of the thermocouples

The measurement of a thermocouple voltage can only be related to a local temperature if the Seebeck coefficient $S_{\rm tc}$ of the thermocouple is known. Here, we calibrate our NiCu-Au thermocouples by determining $S_{\rm tc} = S_{\rm NiCu} - S_{\rm Au}$ in a separate device geometry.

Supplementary Figure 1 shows the set of measurements that we used to determine $S_{\rm tc}$ of a thin NiCu film contacted by Au electrodes. For this, we used a microfabricated Au heater and Au resistive thermometers similarly as described in reference[1]. In our case, we sputtered 30 nm of NiCu in a rectangular shape of 31 µm × 10 µm, as shown in Supplementary Figure 1(a). We calibrated the resistive thermometers by measuring the linear temperature dependence of the 4-probe resistance $R_{\rm Au}$ of the Au bars as shown in Supplementary Figure 1(b), using a global heater in the sample mount. Next, we measured the local resistance at each of the Au bars as a function of a dc current $I_{\rm dc}$ through the microfabricated heater. The dependence is quadratic, because in this temperature range $\Delta R_{\rm Au} \propto \Delta T \propto P \propto {I_{\rm dc}}^2$, where P is the Joule power generated at the heater. A typical measurement with parabolic fit is depicted in Supplementary Figure 1(c). Thus, using the two results above we were able to measure the temperature profile along the NiCu film, as shown in Supplementary Figure 1(d). Finally, measuring the second harmonic response between two different Au bars to an ac

current I_{ac} through the microfabricated heater, we show in Supplementary Figure 1(e) the Seebeck potential difference ΔV induced by the Joule heating. By comparing ΔV with the temperature difference ΔT obtained by the previous resistive thermometry, we extract the Seebeck coefficient $S_{\mathrm{NiCu}} - S_{\mathrm{Au}} = -27.3 \pm 7.2$ μV K^{-1} [Supplementary Figure 1(f)]. We note that this value is in good agreement with values reported earlier[2].

Supplementary Note 2. Analysis of the thermocouple signal

In order to determine the response caused purely by the Peltier effect it is necessary to carefully analyze the thermocouple response to the current applied to the Peltier junction. In Supplementary Figure 2(a) we show the raw data leading to the signal shown in Fig. 3 of the main text. Here we use the same current source configuration (I) as shown in Fig. 2 of the main text. We define the signal V_{34} as the plus and minus of the voltage probe at contact 3 and 4 respectively, and V_{43} as its reverse. The common mode (CM) signal, given by $(V_{34} + V_{43})/2$, is the background that is caused by any potential relative to ground that is present at both voltage probes, which in this case is constant versus V_{g} as it only involves the metallic leads. The true differential mode signal (DM) is then given by $(V_{34} - V_{43})/2$. The plot shows that the DM signal is, minus an offset, the true Peltier signal shown in Fig. 3 of the main text.

Supplementary Figure 2(b) shows similar measurements, for the second configuration (II) described in the main text, with exchanged current source connections. Here, the CM signal is gate dependent because there is a graphene resistance between the Peltier junction and the ground. Its origin can be measured independently by applying a current from contact 2 to 1 and measuring the voltage V_{41} (with contact 1 grounded). The red line in Supplementary Figure 2(b) shows the gate dependence of this 3-probe resistance labeled R_3, multiplied by the common mode rejection ratio CMRR $= 8.9 \times 10^{-6}$ of the electronics, in good agreement with the CM signal.

We performed all our measurements at low frequency $f < 10$ Hz, ensuring a consistent and frequency independent line shape. The remaining (gate independent) background offset can be evaluated by its frequency dependence. Such background signal comes from capacitive coupling. Therefore, the true value of the Peltier signal is found in the 0 Hz limit. In Supplementary Figure 2(c) is a plot of the f dependence of the DM signals for both measure-

ment configurations at $V_{\mathrm{g}} = 0$ V. Using a second order fit to extrapolate to $f = 0$ Hz we thus obtain the background to substract from the raw DM signals of Supplementary Figure 2(a) and Supplementary Figure 2(b), leading to the final signals of Fig. 3 of the main text. Thus, we confirmed the zero-crossing of the Peltier signal, and thereby the cross-over between heating and cooling, at the charge neutrality point. Supplementary Figure 2(d) shows that the CM signal is f independent for both measurement configurations. Note that, as expected, the smaller contributions of CM and capacitive signals are found for configuration (I), so we focus on this configuration for an accurate analysis.

Supplementary Note 3. Low temperature measurement

Thermoelectric coefficients usually have a strong temperature dependence, given they are thermodynamic properties. Therefore, we have repeated the Peltier measurements at liquid nitrogen (LN) temperature. Supplementary Figure 3 compares the measurement as in Fig. 3 of the main text with the same measurement at LN (77 K). At LN we observed a response with a lineshape consistent with that at room temperature (RT), going to more negative values when crossing from the hole to the electron regime, except for an offset of about -0.5 mΩ which lies at the limit of our measurement accuracy. More importantly, the modulation of the signal is about 1 mΩ or one order of magnitude smaller than at RT.

We can understand this strong suppresion of the signal from Eq. 1 of the main text, which leads to $V_{\mathrm{tc}}/I = S_{\mathrm{tc}} \Pi_{\mathrm{gr}} R_{\mathrm{th}}$. Considering a linear temperature dependence for the Seebeck coefficients[1], $S \propto T$, and the second Thomson relation[3], $\Pi = ST$, we deduce a scaling of the form $S_{\mathrm{tc}} \Pi_{\mathrm{gr}} R_{\mathrm{th}} \propto T^3 R_{\mathrm{th}}$. The first factor, T^3, leads to a scaling of $(293\ \mathrm{K}/77\ \mathrm{K})^3 = 55$. An accurate evaluation of R_{th} requires detailed modelling and inclusion of several material parameters, but as a first order approximation we consider $R_{\mathrm{th}} \propto 1/\kappa_{\mathrm{gr}}$. Since κ_{gr} has a fivefold decrease from room temperature to LN[4] our final estimate for the scaling, $V_{\mathrm{tc}}^{RT}/V_{\mathrm{tc}}^{LN} = 55/5 = 11$, is in agreement with the experimental result, further demonstrating the Peltier thermoelectric nature of the measurements.

SUPPLEMENTARY REFERENCES

[1] Zuev, Y. M., Chang, W. & Kim, P. Thermoelectric and Magnetothermoelectric Transport Measurements of Graphene. *Phys. Rev. Lett.* **102**, 096807 (2009).

[2] Bakker, F. L., Flipse, J. & Wees, B. J. v. Nanoscale temperature sensing using the Seebeck effect. *J. Appl. Phys.* **111**, 084306 (2012).

[3] Callen, H. B. The Application of Onsager's Reciprocal Relations to Thermoelectric, Thermomagnetic, and Galvanomagnetic Effects. *Phys. Rev.* **73**, 1349–1358 (1948).

[4] Seol, J. H. *et al.* Two-Dimensional Phonon Transport in Supported Graphene. *Science* **328**, 213–216 (2010).
